# Intensive home treatment compared to inpatient psychiatric treatment: a 36-month follow-up of a propensity-score matched retrospective multicenter cohort study

**DOI:** 10.1186/s12888-026-08062-5

**Published:** 2026-04-16

**Authors:** Konstantinos Nikolaidis, Sandeep Rout, Olaf Hardt, Christoph Richter, Britta Janßen, Jürgen Timm, Andreas Bechdolf

**Affiliations:** 1 Department of Psychiatry, Psychotherapy, and Psychosomatics Incorporating FRITZ am Urban and Soulspace, Vivantes Hospital am Urban and Vivantes Hospital im Friedrichshain, Dieffenbachstr. 1, 10967 Berlin, Germany; 2 Department of Psychiatry and Psychotherapy, Charité Campus Mitte, Charité Universitätsmedizin Berlin, Corporate Member of Freie Universität Berlin, Humboldt-Universität Zu Berlin, Charitéplatz 1, 10117 Berlin, Germany; 3 Department of Psychiatry, Psychotherapy and Psychosomatics, Vivantes Hospital Neukölln, Berlin, Germany; 4 Department of Psychiatry, Psychotherapy and Psychosomatics, Vivantes Hospital Kaulsdorf, Berlin, Germany; 5 Competence Center for Clinical Studies Bremen, Department of Biometry, University of Bremen, Bremen, Germany; 6 German Center for Mental Health – Berlin-Potsdam Site, Berlin, Germany

**Keywords:** Intensive home treatment, Propensity score matching, Psychiatric service utilization, Readmission rates

## Abstract

**Background:**

Intensive Home Treatment (IHT) has been implemented as an alternative to inpatient psychiatric treatment for selected patients requiring acute care. While short-term reductions in inpatient utilization have been reported, evidence on long-term outcomes beyond 12 months—particularly regarding readmissions, treatment days, and broader psychiatric service use—remains limited. This study evaluates the 36-month effectiveness of IHT compared to inpatient treatment (IT) in routine care, focusing on readmission rates, treatment days, and outpatient service engagement.

**Methods:**

We conducted a retrospective propensity-score matched cohort study using routine clinical data from three psychiatric hospitals in Berlin, Germany. Patients receiving IHT were matched (1:1) to patients receiving IT based on age, gender, diagnosis, and prior service utilization. Outcomes included inpatient and combined readmissions (IT, IHT and day clinic), treatment days, time to readmission, and first-time use of the Psychiatric Outpatient Department (POD) over 36 months. Statistical analyses included binary logistic regression, Kaplan–Meier survival analysis with log-rank testing, and non-parametric tests.

**Results:**

263 patients receiving IHT were matched to 263 patients treated with IT, with no statistical differences at baseline between groups. The IHT group had significantly lower inpatient readmission rates (41.1% vs. 55.5%, *p* = 0.001), fewer inpatient readmissions (mean 1.72 vs. 2.02, *p* = 0.005), and fewer inpatient days (48.5 vs. 51.7, *p* = 0.003) compared to IT over 36 months. Time to readmission was longer for IHT (median not reached vs. 610 days for IT, *p* = 0.001). Combined readmission rates (IHT + IT+day clinic) did not differ significantly (61.2% vs. 64.3%, *p* = 0.47). IHT patients were more likely to initiate for the first time a POD treatment (33.5% vs. 24.7%, *p* = 0.035) and had more IHT readmissions (mean 0.85 vs. 0.35, *p* < 0.001).

**Conclusions:**

In this selected cohort of patients deemed suitable for home-based acute care, IHT was associated with fewer inpatient readmissions and a longer time to inpatient readmission over a 36-month follow-up period. Overall acute psychiatric care use (inpatient + IHT) did not differ between groups, suggesting that IHT may not reduce overall acute care need but may shift care from inpatient settings toward home-based treatment.These findings should be interpreted cautiously given the non-randomized study design and the likelihood of residual selection bias. Limitations include restricted generalizability to rural areas and lack of clinical symptom data. Further multi-centre studies are needed to confirm these results regarding long-term effects across diverse healthcare systems.

**Trial registration:**

German Clinical Trials Register (DRKS), DRKS00036833. Registered May 21, 2025, https://www.drks.de/search/de/trial/DRKS00036833/details.

## Background

Intensive Home Treatment (IHT) approaches—such as crisis resolution teams—have been implemented in many mental health systems as effective alternatives to inpatient care for individuals requiring intensive, community-based psychiatric care [1]. These services provide intensive, multidisciplinary psychiatric support directly in patients’ homes, aiming to reduce the need for hospital admissions. Over the years, findings have suggested that IHT can reduce inpatient service utilization [2].

However, earlier studies only partially addressed the broader use of psychiatric services—such as day clinics and outpatient support—which play a vital role in contemporary mental health care systems. More recently, a prospective quasi-experimental multicenter trial conducted across 10 sites in Germany [3], provided new evidence by examining the newly implemented German IHT model. First findings demonstrated significantly lower inpatient and combined readmission—defined to include inpatient care, IHT, and day clinic utilization— among patients receiving IHT compared to those treated in inpatient settings [4]. In addition, IHT was associated with higher treatment satisfaction and greater involvement in shared decision-making [5], and was shown to be cost-effective at a slightly higher acceptability probability than inpatient treatment [6].

Furthermore, a retrospective pilot study on the German IHT model with matched pairs showed an effect of significantly rising number of first-time treatments in the Psychiatric Outpatient Department (POD) following the IHT treatment in comparison to inpatient treatment at a 12-month-follow-up [7].

This German-specific form of IHT, known as inpatient equivalent home treatment (IEHT), was allowed to start in 2018 after legal framework was clarified in 2016. Thus, it was made available to German psychiatric hospitals through reimbursement by public health insurance. It shares several core features with established home treatment (HT) models like the British, Norwegian, Spanish, Swiss and Dutch [1, 8–12], including regular home visits, small caseloads, comprehensive psychiatric and medical assessments conducted at home, shared responsibility for both medical and social care, intensive support, involvement of family members or carers, and the development of crisis plans Nevertheless, unlike most other HT models, IHT in Germany operates under strict regulatory requirements [13]. Teams are required to conduct daily home visits, provide at least two weekly psychiatric consultations, and hold weekly multiprofessional meetings to ensure comprehensive care. Compliance with these procedural standards is directly tied to reimbursement, fostering quality assurance and consistency across sites but limiting flexibility for local adaptation This consistent standardization across sites creates favorable conditions for rigorous and high-quality evaluation of this model. Importantly, eligibility for IHT in routine care is typically restricted to a subset of patients presenting with acute psychiatric symptoms. Regulatory and clinical requirements—such as a stable home environment, consent of household members, sufficient cooperation, and manageable risk profiles—mean that only a proportion of patients presenting for acute inpatient care can realistically be considered for IHT. Consequently, outcomes observed in IHT cohorts may reflect both treatment effects and pre-existing differences in patient suitability.

This growing body of evidence supports IHT as a viable and effective alternative to inpatient treatment (IT), particularly when implemented in routine care. Nevertheless, most previous studies have examined relatively short follow-up periods—typically 6, 12 [8], or occasionally 24 months [12]. Data on long-term outcomes beyond the first year remain limited, especially concerning readmissions, cumulative treatment days, and the combined use of psychiatric services, including IT, IHT, and day clinic (DC). These outcomes are crucial for evaluating the sustainability and systemic effects of IHT.

To help fill these gaps, the present study investigates the long-term effectiveness of IHT in routine care by analysing a large retrospective matched cohort over a 36-month follow-up period. Specifically, the study focuses on inpatient readmission rates as well as the broader utilization of psychiatric services in a real-world clinical setting, the inpatient and combined readmission rates, the total treatment days and the effect on the utilization of outpatient treatment services. Given that survival analysis offers a more comprehensive, flexible, and clinically meaningful framework for predicting and managing hospital readmissions compared to simple readmission rates, we also planned to assess the time to readmission.

## Methods

### Study setting and data source

This study is based on routine controlling data and electronic patient records from the Vivantes Departments of Psychiatry, Psychosomatics, and Psychotherapy at the “Am Urban” (KAU), “Neukölln” (KNK), and “Kaulsdorf” (KHD) hospitals. The data cover patients treated between January 1, 2020, and December 31, 2020. No direct interviews or surveys were conducted. All clinics are part of a state-owned hospital group, serving approximately 1.2 million residents across three districts of Berlin.

Two of the main contributing centres—KNK and KAU—are comparable in structure and service delivery. Following the prior implementation and evaluation of HT and intensified outpatient care as part of a pilot project under § 64b SGB V, IHT was introduced in 2018 with an initial capacity of seven treatment slots. Both clinics offer a wide range of psychiatric treatment services, including specialized programs for young people in psychological crises [14–16], as well as job coaching based on the Individual Placement and Support (IPS) model [17], have been offering IHT since 2018 and maintained established IHT teams throughout the observation period. Though working in different models. In 2020, KNK operated approximately 14 IHT treatment places, while KAU initially had 14 places and increased to 20 places in November 2020. Regular IHT services at KHD officially began on October 6, 2020, with initially 7 places offered, bringing the total number of IHT cases in 2020 to 42. The KHD is comparable in size and structure to the KAU, and it is subspecialized and mandatory care clinic for the Berlin district of Marzahn-Hellersdorf.

### Intervention and control treatment

IHT is a psychiatric treatment delivered at the patient’s home by a multidisciplinary group—including psychiatrists, psychologists, psychiatric nurses, social workers, and other professionals. The treatment protocols for IHT in Germany, called inpatient equivalent home treatment (IEHT), have been thoroughly defined by the umbrella organization representing all German social health insurance providers and the German Hospital Federation [13]. To qualify for reimbursement by German statutory health insurers, certain criteria must be met: there must be a formal indication for acute inpatient treatment, a psychiatrist must evaluate the patient’s suitability for home treatment (including the availability of a permanent home environment and sufficient privacy), a written treatment plan must be established, the team must be available every day of the week—round the clock (outside regular hours, on-call psychiatrists at the emergency department cover nighttime), and at least one in-person contact must occur each day. The team must also hold weekly meetings to review each patient in detail, and every contact (with exact time and content) must be documented.

The IHT team handles all psychiatric and physical health care needs—covering diagnostic assessments, medication management, psychotherapy, and social support. An individualized needs assessment is conducted during the initial days, which forms the backbone of a tailored treatment plan. This plan outlines goals and interventions, such as medication, psychotherapy, skills training, daily or therapeutic activities, and family or caregiver involvement. Interventions are adjusted daily to meet patient needs, and in-person contacts may occur at home, in the hospital, or in any location chosen by the patient. IHT is not restricted to specific diagnostic or demographic subgroups but is available to all patients who meet the criteria for acute inpatient psychiatric treatment.

The inpatient care was conducted in accordance with the established quality and organizational standards for psychiatric hospitals in Germany.

Treatment allocation followed standardized clinical protocols across all participating psychiatric facilities. All patients met diagnostic criteria for acute psychiatric admission before treatment assignment. IHT eligibility was determined through a two-tier assessment combining regulatory requirements and clinical evaluation. Regulatory requirements included: (1) availability of permanent and suitable home environment for therapeutic activities, (2) written consent from all adult household members, (3) absence of child welfare concerns, and (4) residence within the hospital’s catchment area. Clinical evaluation assessed risk factors, including treatment adherence history, to determine whether patients could safely receive intensive home-based care. Given limited IHT capacity (14–20 treatment slots per facility compared to larger inpatient capacity), allocation decisions prioritized patients who met both regulatory requirements and demonstrated suitability for home-based treatment to ensure effective treatment delivery and appropriate resource utilization. As a result, treatment allocation was not random and inherently favored patients perceived as clinically and socially suitable for home-based care, which may have contributed to systematic differences between treatment groups that could not be fully adjusted for in retrospective analyses.

### Study sample

This is a retrospective cohort follow-up study with a group receiving IHT and a matched group of IT users. The IHT group includes all patients who received IHT between January 1, 2020, and December 31, 2020, at KAU, KNK, or KHD. For each patient, the index treatment episode (i.e., the first IHT episode within the observation period) was identified, and the last day of this episode served as the starting point for a 36-month follow-up period. The IT group was drawn from patients who were admitted for full IT during the same period at the same clinics.

Patients were excluded if they:

- Received care under the regulation of the German Social Code Book V, Section 64b model project due to insurance status.
- Were private patients or self-payers.
- Had an index treatment period shorter than five days.
- Had no primary psychiatric diagnosis (F-code) according to ICD-10.
- Were not living in the catchment area of the reference hospital, as being an exclusion criterion for providing IHT from the reference hospitals in general.

### Matching procedure

To ensure comparability between groups, a propensity score matching (PSM) approach was applied. Based on logistic regression analyses of data from the preceding year, a propensity score (PS) was calculated for each patient using the variables: age, gender, primary psychiatric diagnosis (FX), and the number of full IT or IHT received at the clinic within the last two years.

Each IHT case was matched to a control case from the IT group within the same diagnostic group (FX) using the nearest neighbour method without caliper [18]. The order of IHT patients was randomized, and the pairing was defined by the smallest sum of squared PS differences. This resulted in two matched cohorts: 263 patients in the IHT group and 263 in the IT group. A similar matching framework was also used in the multicentre, prospective AKtiV study [19]. In the present retrospective routine-data study, however, baseline comparability could only be assessed using the limited set of routinely recorded variables available across sites.

The matching process was carried out through the following steps:

- Stratified matching within diagnostic subgroups (FX).
- Nearest neighbour matching based on PS proximity.
- Iterative pairing without replacement, with randomized order of IHT group patients.
- Evaluation of matches by the mean PS difference across all pairs.
- Selection of the matching set with the smallest total squared PS difference.

Because this was a retrospective routine-data study, baseline comparability could only be assessed using the variables that were consistently available across all participating sites. These were age, gender, diagnosis group, and prior acute psychiatric service utilization, which therefore constituted the propensity score model. No harmonized data on symptom severity, psychosocial functioning, housing context, or social support were available.

### Outcomes

Over a 36-month observation period following discharge from the index treatment, the following outcomes were analysed:

- IT readmission rate (primary outcome): Proportion of patients readmitted to IT psychiatric care after the initial treatment episode.
- Combined readmission rate (including IT, IHT, and DC settings): Proportion of patients readmitted to any structured psychiatric care setting, including IT, IHT, or DC treatment.
- Number of full inpatient treatment readmissions: Total number of times patients were readmitted to full inpatient psychiatric care during the follow-up period.
- Number of treatment days during IT readmissions: Total number of days patients spent in IT after being readmitted.
- Number of IHT readmissions: Total number of times patients were readmitted to IHT.
- Number of treatment days during IHT readmissions: Total number of days patients received IHT after being readmitted.
- Number of DC treatment readmissions: Total number of times patients were readmitted to DC treatment.
- Number of treatment days during DC readmissions: Total number of days patients spent in DC after being readmitted.
- Number of first-time treatments in the POD following the initial treatment: Number of patients who initiated treatment in the POD after completing their initial (index) treatment.
- Time to readmission: Time to readmission was defined as the number of days between the discharge date of the initial (index) admission and the date of the subsequent IT psychiatric readmission.

These indicators provide a comprehensive evaluation of long-term healthcare utilization and support the assessment of sustained treatment effectiveness and service continuity in psychiatric care.

The secondary outcomes “number of coercive measures”, “cumulative duration of coercive measures within 36 months after the end of the index treatment”, as well as “premature treatment termination during the index treatment”, as detailed in the study protocol, could not be assessed due to technical limitations in the hospital information systems across all participating clinics. These outcomes will therefore be reported separately in a forthcoming publication focusing exclusively on the KAU site population.

For the comparison of treatment days during the index stay as well as treatments after the index stay and for the number of readmissions (IT, IHT, or DC), means with standard deviations were calculated. The dichotomous (yes, no) variable of readmission (IT, IHT, or DC) was defined as one admission within 36 months after discharge from the index treatment into the same treatment modality, with a minimum length of stay of one day. The proportion refers to the percentage of patients who experienced at least one such readmission within that time frame.

The dichotomous (yes, no) variable of combined readmission was defined as one admission within 36 months after discharge from the index treatment into any of the three treatment modalities (IT, IHT, or DC), with a minimum length of stay of one day.

A first-time treatment in the POD of the clinic was defined as the utilization of POD services if no POD services of the clinic had been utilized in the previous two years.

Time to readmission was calculated by subtracting the index discharge date from the IT readmission date, resulting in the total days until IT readmission.

### Data analysis

Sociodemographic and treatment-related data from all matched patients were extracted from the internal documentation system. Data were analysed using R (Version 4.3.0) [20]. Descriptive statistics for categorical variables are reported as absolute and relative frequencies (n, %), and continuous variables as means (M) and standard deviations (SD). The Shapiro-Wilk test was applied to assess normality of continuous variables. Group comparisons were conducted using the Mann-Whitney U test for continuous variables and the chi-squared test for categorical variables. To examine factors associated with the full inpatient readmission within 36 months, a sensitivity analysis using binary logistic regression was conducted. The model included readmission status (yes/no) as the dependent variable and treatment group (IHT vs. IT), along with all possible variables showing significant between-group differences in the baseline comparison, as independent variables. Time to inpatient readmission was analysed using Kaplan–Meier survival curves, which were used to estimate the probability of remaining free from inpatient readmission over the 36-month follow-up period in both treatment groups. Group differences were assessed using the log-rank test.

Although the data were drawn from multiple participating centres, we did not perform centre-specific analyses. The aim of this study was not to examine differences between individual sites, but rather to gain a comprehensive, system-level understanding of the effectiveness of IHT in real-world psychiatric care across a broader population.

As a post hoc exclusion sensitivity analysis, we re-ran the main outcome analyses after excluding five control-group cases that were subsequently identified on chart review as lacking permanent housing at the time of index admission. Because stable housing is a practical prerequisite for the delivery of IHT in routine care, these cases may not have been realistically eligible for IHT and may therefore have introduced additional structural imbalance between groups. This analysis was conducted to assess whether the main findings were robust to exclusion of these potentially non-comparable cases.

### Sample size calculation

The sample size in the retrospective data collection depends on the one hand, on the availability and completeness of the retrospective data and, on the other hand, on the expected effect size. A prospective study [4] with a 12-month follow-up and a sample size of 400 patients (200 per group) showed that IHT was associated with a significantly lower rate of full inpatient readmissions compared to traditional full inpatient treatment (31.12% vs. 49.74%; mean difference: 18%).

However, for a planned 3-year follow-up, it is expected that the difference between the groups in terms of full inpatient readmission rates may be smaller. This suggests that a larger sample size than the 400 patients used in that study will be required to statistically detect even smaller differences over the longer observation period. A new sample size calculation was therefore necessary to account for the changed assumptions regarding a longer follow-up and a smaller effect size.

Considering the possible available data in the hospital information system (HIS), a sample size of approximately 350 patients in the IHT group could be feasible. A comparable control group with 350 patients from the full inpatient treatment context will be selected using propensity score matching (PSM). With a sample size of 350 patients per group, a power of 80%, and a two-sided chi-squared test with an alpha error of 5%, an effect size of up to 0.21 can be detected. This would correspond to a reduction in effect size of up to approximately 45% compared to the prospective 1-year follow-up study.

## Results

### Sample and matching procedure

Between January 1, 2020, and December 31, 2020, a total of 314 patients were admitted to intensive home treatment (IHT). Of these, 51 patients (16%) were excluded because their index treatment duration was fewer than five days. No exclusions were made due to missing psychiatric (F-code) diagnoses or residency outside the hospital’s catchment area. Thus, 263 IHT patients (84%) were available for matching.

During the same period, 5,241 patients received full inpatient psychiatric treatment. Of these, 2,757 patients (53%) were excluded: 76 (1.5%) due to missing F-code diagnoses, 1,694 (32.3%) due to treatment duration of fewer than five days, 881 (16.8%) due to residency outside the catchment area, and 106 (2.0%) due to duplicate records with participants from the group of the IHT patients. Consequently, 2,484 inpatient cases (47%) were eligible for propensity score matching.

Matching was conducted at a 1:1 ratio based on clinically relevant covariates using propensity scores. As no calliper was applied, the small number of matched pairs (*n* = 5**)** with a propensity score difference greater than 10 points were not excluded. All remaining matched pairs had differences below 0.8. In total, 263 matched pairs (*n* = 526 patients) were included in the final analysis.

No statistically significant differences were found between the two groups regarding the variables relevant to the propensity score matching. However, prior POD utilization differed significantly between groups. The values of all variables relevant to the PSM procedure can be found in Table 1.

**Table 1:** Propensity score variables of the participants in the IHT compared to the participants in IT group at recruitment

Propensity score variables	IHT group (*n* = 263)	IT group (*n* = 263)	*p* value
Gender female; n (%)	162 (61.6)	150 (57.0)	0.3289^a^
Age in years; mean (SD)	47.30 (17.36)	48.22 (17.23)	0.469^b^
Number of IHT or IT in the 2 years prior to the start of the index treatment; mean (SD)	1.75 (2.26)	1.46 (2.21)	0.05^b^
Main diagnosis on admission to index treatment	1:1 matching		1.000 ^a^
F0X; n (%)	6 (2.3)	6 (2.3)	
F1X; n (%)	5 (1.9)	5 (1.9)	
F2X; n (%)	100 (38.0)	100 (38.0)	
F3X; n (%)	111 (42.2)	111 (42.2	
F4X; n (%)	27 (10.3)	26 (9.9)	
F5X; n (%)	1 (0.4)	1 (0.4)	
F6X; n (%)	13 (4.9)	14 (5.3)	

IHT = intensive home treatment; IT = inpatient treatment; SD = standard deviation; n = frequency, a = chi-squared test; b = Wilcoxon test

Prior to the index treatment POD utilization differed significantly between groups. In the IHT group, 127 of 263 patients (48.3%) had at least one POD use prior to the index admission, compared with 97 of 263 patients (36.9%) in the inpatient group. This difference was statistically significant (Fisher’s exact test, *p* = 0.010).

Following the reviewers’ comments, we identified five matched control-group cases that lacked permanent housing at the time of index admission based on chart review. As stable housing is a practical prerequisite for IHT in routine care, these cases may not have been realistically eligible for IHT. We addressed this issue in a post hoc exclusion sensitivity analysis by excluding these five control cases and their matched IHT counterparts, resulting in a reduced matched sample of 258 pairs (*N* = 516).

### Results of the index treatment and 36-month follow-up

The average duration of the index treatment was 31.80 days (SD = 24.18) in the IHT group and 33.02 days (SD = 35.30) in the IT group, with the difference being statistically significant (*p* = 0.016, Wilcoxon test).

Within the 36 months following discharge, 108 participants in the IHT group (41.1%) experienced at least one full inpatient readmission. In contrast, 146 participants in the IT group (55.5%) were readmitted at least once. The inpatient readmission rate was significantly lower in the IHT group by approximately 14.4% (*p* = 0.001; chi-squared test), indicating a meaningful difference between groups.

A sensitivity analysis using logistic regression for the primary outcome (readmission within 36 months), including index treatment duration and treatment group (IHT vs. IT), confirmed that the treatment effect remained statistically significant even after adjusting for index treatment duration (*p* < 0.001).

Because prior POD utilization before the index treatment differed significantly between groups at baseline, we performed a sensitivity analysis using binary logistic regression for the primary outcome (any inpatient readmission within 36 months), including treatment group and prior POD utilization as predictors. After adjustment, the IT group remained associated with higher odds of inpatient readmission within 36 months than the IHT group (OR = 1.98, 95% CI 1.39–2.83, *p* < 0.001), corresponding to lower odds in the IHT group (inverse OR = 0.51). Prior POD utilization was also independently associated with higher odds of inpatient readmission (OR = 2.20, 95% CI 1.54–3.16, *p* < 0.001). The combined readmission rate within 36 months after discharge was 61.2% in the IHT group and 64.3% in the IT group. Although the intervention group showed a lower readmission rate by about 4.2% points, this difference was not statistically significant (*p* = 0.47; chi-squared test).

The number of inpatient readmissions during the 36-month follow-up was also significantly higher in the IT group compared to the IHT group (IT M = 2.02, SD = 3.26; IHT: M = 1.72, SD = 3.70; *p* = 0.005; Wilcoxon test).

Additionally, the number of inpatient treatment days during the follow-up period was significantly higher in the IT group (M = 51.66, SD = 94.44) than in the IHT group (M = 48.47, SD = 117.64; *p* = 0.003; Wilcoxon test).

Participants in the IHT group were significantly more likely to be admitted to IHT again during the 36-month follow-up than those in the IT group (IHT group: M = 0.85, SD = 1.87; IT group: M = 0.35, SD = 1.31; *p* < 0.001; Wilcoxon Test). They also spent significantly more days in IHT (IHT group: M = 21.55, SD = 44.39; IT group: M = 7.41, SD = 31.65; *p* < 0.001; Wilcoxon Test).

Participants in the IHT group were significantly more likely to receive first-time treatment in the hospital’s POD during the 36-month period following the index treatment. A total of 88 IHT group participants (33.5%) initiated new POD treatment, compared to 64 in the IT group (24.7%), representing a significantly higher rate of new POD treatment in the IHT group by nearly 12% (*p* = 0.035; chi-squared test).

No significant differences between the groups were observed regarding combined readmissions, the number of DC readmissions, or the number of DC treatment days during the 36 months following discharge from the index treatment.

A detailed overview of the utilization of psychiatric services within 36 months after initiation of the index treatment in the IHT group compared to the IT group can be found in Table 2.

**Table 2:** Utilization of psychiatric services within 36 months after index treatment

	IHT Group	IT Group	*p* value
IT readmission rate; *n* (%)	108 (41.1%)	146 (55.5%)	0.001^a^
Total number of IT readmissions; mean (SD)	1.72 (3.70)	2.02 (3.26)	0.005^b^
Total number of IT treatment days follow-up cases; mean (SD)	48.47 (44.39)	51.66 (94.55)	0.003^b^
Combined readmission rate; n (%)	161 (61.2%)	169 (64.3%)	0.471^a^
Total number combined readmissions; mean (SD)	2.86 (5.27)	2.70 (4.40	0.741^b^
Total number combined treatment days follow-up cases; mean (SD)	81.60 (141.95)	70.74 (118.76)	0.950^b^
Number of IHT readmissions; mean (SD)	0.85 (1.87)	0.35 (1.31)	0.000^b^
Total IHT treatment days of follow-up cases; mean (SD)	21.55 (44.39)	7.41 (31.65)	0.000^b^
Number of DC readmissions; mean (SD)	0.29 (0.84)	0.33 (0.77)	0.133^b^
DC treatment days of follow-up cases; mean (SD)	11.59 (34.24)	11.67 (27.17)	0.223^b^
First-time treatment in the POD after the Index Treatment; n (%)	88 (33.5%)	65 (24.7%)	0.035^a^

IHT = intensive home treatment; IT = inpatient treatment; POD = Psychiatric Outpatient Department; DC = Day clinic; n = frequency; SD = standard deviation; a = chi-squared test; b = Wilcoxon test; combined treatment = inpatient treatment + intensive home treatment + day clinic treatment

**Fig. 1:**
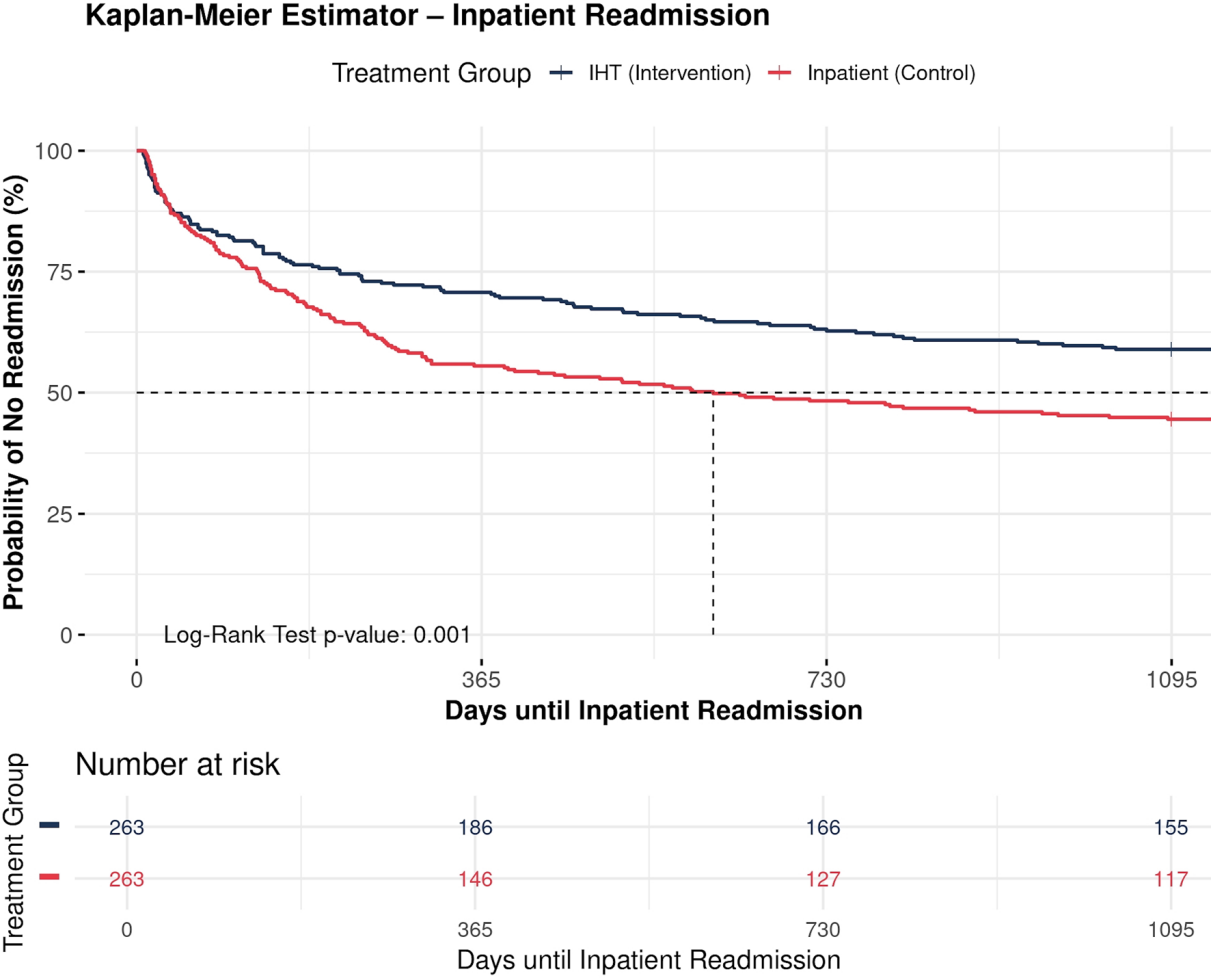
Kaplan-meier estimator of inpatient readmission 36-months after the index treatment. IHT = intensive home treatment; IT = inpatient treatment

Participants in the IHT group showed a significantly longer time to IT readmission compared to the IT group over the 36-month (1,095-day) follow-up period. The log-rank test showed a significant difference between the groups (χ² (1) = 10.5, *p* = 0.001), indicating better survival (longer readmission-free time) in the IHT group. The median time to readmission in the IHT group was not reached within the 1,095-day follow-up period (median = 1,197 days; the upper confidence limit is not estimable), suggesting that more than half of the IHT group participants remained readmission-free at study end. In contrast, the IT group had a median time to readmission of 610 days (95% CI: 357 to 1,212 days), indicating that half of the IT group participants experienced readmission by approximately 20 months. The Kaplan-Meier survival curves (Fig. 1) show a consistently higher probability of avoiding full inpatient readmission over time for the IHT group compared to the IT group.

By the end of the follow-up period, the cumulative probability of remaining without readmission was markedly higher in the IHT group.

In the post hoc exclusion sensitivity analysis excluding five control-group cases lacking permanent housing at index admission and their matched IHT counterparts (258 pairs; *N* = 516), the overall pattern of findings remained unchanged. The primary outcome remained statistically significant (41.1% vs. 55.0%, *p* = 0.002), whereas combined readmission remained non-significant.

## Discussion

Consistent with previous research [4, 7, 21] over a 12-month follow-up, IHT was associated with significantly fewer inpatient readmissions, fewer inpatient days, and a reduced number of total inpatient episodes over a 36-month follow-up period. The significantly longer readmission-free survival in the IHT group, with the median time to readmission not being reached within the observation period, suggests a robust and lasting effect of IHT on prevention of inpatient readmission. In contrast, the IT group reached the median time to readmission at 610 days, highlighting a substantially higher risk of rehospitalization over time. These results extend previous findings by suggesting the benefits of IHT are not limited to short-term outcomes but may be sustained over the longer term, indicating that associations between IHT and reduced inpatient utilization can persist beyond the short term in selected patient populations. Taken together, the findings suggest that IHT may represent a feasible and potentially effective alternative to conventional IT for selected individuals requiring intensive psychiatric care [4].

The findings of this study apply to a clinically and socially selected subgroup of patients with acute psychiatric disorders. Patients receiving IHT were required to have a suitable home environment, provide informed consent, and demonstrate sufficient cooperation and stability to allow safe treatment at home. Consequently, these results are unlikely to generalize to involuntarily treated, homeless, or high-risk populations that constitute a substantial proportion of acute psychiatric admissions in many jurisdictions.

With respect to the routinely recorded baseline variables available in this retrospective dataset—namely age, gender, diagnosis group, and prior acute psychiatric service utilization—the two groups were similar after propensity score matching. However, no additional harmonized baseline data (e.g., symptom severity, psychosocial functioning, housing context, or social support) were available across sites, and unmeasured differences may therefore remain. The study population mainly comprised adults with severe mental disorders—most frequently affective (F3x) and psychotic (F2x) conditions—which together accounted for approximately 80% of all included cases. With a mid-adult age distribution (mean age 47 years) and balanced gender ratio (61.6% female), this cohort reflects the typical target group of inpatient-equivalent home treatment (IHT) in Germany. These findings are consistent with previous German studies employing propensity score matching [4, 7], which reported similar diagnostic and demographic patterns and mirror international research from Switzerland [12] and the Netherlands [8]. Across these contexts, IHT programs consistently target adults with severe affective and psychotic disorders—typically aged in their forties—who retain sufficient stability and social support to engage in structured, community-based treatment.

While differences in outcomes were observed following the index episode, these findings cannot be causally attributed to the treatment modality alone, as residual confounding and selection effects are likely in this non-randomized design. This is despite the fact that the two groups did not differ statistically with respect to key demographic and clinical variables, such as age, gender, primary psychiatric diagnosis, and the number of prior IT or IHT treatments in the preceding two years.

There were significant differences in the duration of the index treatment, with substantially shorter treatment episodes for the IHT group compared to the IT group. This finding aligns with previous results on the German model of IHT from a monocentric study conducted in the same clinic [7] but not with outcomes from the multicentre study [19], which may reflect procedural differences in the study centres—located in a metropolitan area—compared to the multicentre sample, which also included rural areas and small towns. Nevertheless, a sensitivity analysis using logistic regression showed that the effect of the treatment group on the primary outcome remained significant even after adjusting for the only significant predictor, “duration of index treatment”. While the baseline duration of treatment differed between the IHT and IT groups, the length of the initial (index) hospitalization is not necessarily a direct indicator of symptom severity. In our context, longer inpatient stays may reflect a variety of factors, including differences in treatment effectiveness, organizational or structural aspects of inpatient care, or discharge planning processes.

Despite earlier findings from a prospective non-randomized trial at the 12-month follow-up [4], the current study with a 36-month follow-up could not confirm that IHT leads to a sustained reduction in combined readmissions—including IT, IHT, and DC readmissions. Comparable long-term shifts in service utilization have also been reported in other European settings, including a retrospective cohort study from Spain, which found sustained reductions in inpatient care and increased reliance on community-based services following the implementation of intensive home treatment [22]. From a service utilization perspective, the absence of differences in combined readmissions suggests that IHT does not reduce overall need for acute psychiatric care, but rather redistributes care away from inpatient settings.

However, participants in the IHT group utilized IHT significantly more often and spent more days in IHT during the 36 months following discharge compared to participants in the IT group. Based on these results—and in the absence of further data on psychosocial functioning or symptom severity—it can be concluded that participants in both groups received psychiatric care to a comparable extent during the 36-month follow-up period. From a service-utilization perspective, these findings suggest that overall acute psychiatric care use was similar between groups, although causal inference regarding comparative effectiveness is limited by residual confounding in this non-randomized design. From a healthcare resource perspective, the findings are more consistent with a redistribution of care away from inpatient settings than with a reduction in total acute care need. This finding is in line with results from a previous monocentric study [7] conducted in one of the participating study hospitals, suggesting that the observed patterns may be partly attributable to specific procedural characteristics of the study participating clinics, or of the psychiatric care in metropolitan areas and the specific catchment area of the study clinics [15, 16]. In these settings, a broader range of alternative services to inpatient treatment is available, which may enable patients to substitute inpatient care with other forms of psychiatric service utilization, which are more self-determined and leave patients with more individual freedom than IT. This interpretation aligns with the above discussed findings regarding the shorter duration of index treatment in the IHT group, further reflecting structural and procedural differences between urban study centres and the more diverse settings—including rural areas—captured in previous multicentre studies.

While the study’s implementation within routine care settings strengthens its ecological validity and practical relevance, the exclusive focus on three urban centres in Berlin limits the generalizability of these findings to non-urban or rural areas. Service availability, team structure, and community support networks may differ substantially outside metropolitan regions, potentially influencing treatment feasibility and outcomes. Future studies should therefore examine whether similar benefits of IHT can be replicated in rural or semi-urban contexts, where mental health infrastructure and resource accessibility are more constrained.

A particularly relevant finding of this study and a replication of findings on 12-month follow-up [7] is the higher likelihood of patients in the IHT group to engage for the first time with outpatient psychiatric services—specifically the POD—after the index treatment. Although prior outpatient psychiatric utilization differed between groups, this difference reflects patterns of service engagement before the index admission rather than systematic preselection based on illness severity. Importantly, when analyses were restricted to patients without outpatient psychiatric treatment in the two years preceding the index admission, initiation of outpatient care during follow-up remained more frequent in the IHT group. This finding suggests that intensive home treatment may facilitate improved linkage to outpatient psychiatric services after acute care.

Thus, IHT may contribute not only as a substitute for hospitalization during acute episodes but also as a facilitator of long-term engagement with community-based psychiatric services. This finding supports the hypothesis that home-based treatment may contribute to more continuous, less disruptive care trajectories by fostering stronger integration into outpatient treatment systems.

Although this study did not include a formal economic evaluation, it highlights important aspects of clinical effectiveness and service utilization. Future research should build on these findings by examining the resource implications and cost-effectiveness of IHT compared to inpatient care. Given that Germany has the second-highest number of psychiatric hospital beds in Europe in 2021, with 131 beds per 100,000 inhabitants [23], there is not only potential but indeed a necessity to implement such flexible and resource-optimizing treatment models like IHT to better address current and future demands on mental health services.

These findings suggest also that delivering psychiatric care directly in patients’ homes, even during acute treatment phases, is not only feasible but also offers multiple benefits, not only by reducing the likelihood of future hospitalizations but additionally, the accessibility of the treatment team, collaborative communication on equal footing, and close coordination with other community-based services may play a key role in supporting sustained outpatient care.

However, the effective implementation of IHT cannot stand alone; it requires being embedded within a pluralistic and flexible network of psychiatric services in the community. IHT should be integrated alongside a variety of alternative treatment options—such as outpatient clinics, day treatment programs, crisis services, and supported housing—allowing users to select the care best suited to their needs. This approach enhances therapeutic effectiveness while preserving patient autonomy and choice, essential for sustainable, person-centred mental health care.

The present findings contribute to the growing international evidence supporting IHT as an effective and sustainable alternative to inpatient psychiatric care. Consistent with studies from Europe [8, 12, 21], and our results show that structured, multidisciplinary home-based care can reduce hospital readmissions and support continuity of care. Extending previous 12-month studies, the current 36-month follow-up provides evidence of long-term effectiveness. The German IHT model, with its mandatory daily visits, psychiatrist-led teams, and standardized procedures, differs from more flexible international models (e.g., UK Crisis Resolution Teams) but shares key components—daily contact, small caseloads, and close integration with community services—that appear central to effectiveness across countries. However, successful implementation depends not only on clinical model fidelity but also on contextual factors. Therefore, transferring IHT internationally requires careful local adaptation rather than direct replication. Key prerequisites include accessible community services, logistical capacity for daily home visits, trained multidisciplinary teams, sustainable funding, and cultural acceptance of home-based acute care.

Future research should focus on rural and diverse healthcare settings, examine minimum infrastructure requirements, and include standardized, patient-centered outcomes. While our findings support IHT’s potential globally, they primarily apply to metropolitan systems with strong service networks. Broader implementation should proceed through pilot programs and rigorous evaluation to ensure contextual fit and sustainability.

While our study demonstrates the sustained benefits of IHT, understanding why these effects occur requires examining the interplay of therapeutic, structural, and contextual mechanisms that distinguish home-based from hospital-based acute psychiatric care.

Delivering treatment within the patient’s home fundamentally reshapes therapeutic dynamics. When clinicians enter as guests rather than authorities, traditional power hierarchies are softened, fostering collaboration, mutual respect, and trust. This relational shift likely contributes to the higher shared decision-making scores observed in the AKtiV trial [5]. Patients retain greater control over their environment and can negotiate treatment terms more equitably—conditions known to strengthen the therapeutic alliance, a robust predictor of clinical outcomes across psychiatric interventions. Qualitative research further emphasizes how relational parity and mutual respect within the home setting enhance engagement and alliance formation [24]. Unlike hospitalization, which removes individuals from their everyday contexts, IHT preserves continuity in relationships, roles, and routines. Maintaining participation in familiar environments appears to facilitate functional recovery and integration into ongoing care. The higher rate of outpatient follow-up among IHT patients suggests that this contextual continuity supports smoother transitions to community-based treatment. Practicing coping strategies in real-world settings may further enhance their relevance and durability beyond the acute phase. Because IHT requires household consent and frequently involves cohabitants in treatment, care naturally extends to the broader social system. This systemic approach—addressing crises within their relational context rather than isolating the patient—can target maintaining factors often overlooked in inpatient settings. Active involvement of family members or cohabitants transforms them from peripheral visitors into participants in recovery, fostering shared understanding, early recognition of relapse signs, and more stable home environments [24].

Although the German IHT mandates daily contact, face-to-face sessions are typically shorter than inpatient supervision. This apparent paradox underscores that quality and context of contact may outweigh duration. Purposeful, goal-oriented sessions delivered in the patient’s own environment may foster more focused therapeutic engagement than passive observation in institutional settings.

Treating acute crises within the home may also mitigate stigma, identity disruption, and institutional dependency associated with hospitalization. Remaining in one’s environment reinforces autonomy, self-efficacy, and continuity of identity—factors that may help explain the extended readmission-free survival observed in our study. Moreover, the German IHT framework—with its mandated daily visits, regular psychiatric consultation, and multidisciplinary coordination—offers a balance of predictable structure and contextual flexibility. This combination of consistency and adaptability likely enhances both perceived safety and therapeutic coherence, aligning with best-practice guidance for complex interventions [4]. Taken together, these mechanisms—enhanced therapeutic alliance, preserved autonomy, systemic engagement, contextualized treatment, and structured flexibility—likely operate synergistically rather than independently. Nonetheless, they remain hypotheses requiring empirical validation. Future research should combine quantitative and qualitative methods to clarify which mechanisms are universal versus context-specific, and to identify which patient subgroups benefit most from particular features of home-based acute care.

Beyond therapeutic mechanisms, organizational structures also play a crucial role in shaping the feasibility, scalability, and outcomes of IHT. The heterogeneity of IHT organizational models across countries presents both challenges and opportunities for comparative research. Recognizing these structural differences is essential when interpreting international outcomes. In Germany, IHT is characterized by a high degree of formalization and standardization, whereas other European models operate with broader structural variation, often emphasizing rapid response and short-term crisis intervention rather than daily mandatory visits. These models differ in caseloads, staffing composition, and hours of operation, reflecting contextual differences in healthcare systems, geography, and service philosophy.

To enable meaningful international comparison, future studies should adopt harmonized reporting criteria, including caseload size, contact frequency, team composition, service accessibility, and coverage area. Standardizing these metrics alongside clinical outcomes will clarify which organizational elements are essential for effectiveness and which can be flexibly adapted to local contexts.

## Limitations

This study has certain limitations, particularly regarding the external validity of the results. Although conducted across three centers, all were located within metropolitan Berlin. Consequently, the findings primarily reflect outcomes achievable in urban contexts and may not be directly generalizable to rural or smaller-town settings, where service structures and resource availability differ. Most importantly, this study is subject to confounding by indication, as treatment allocation depended on clinical judgment and regulatory eligibility rather than random assignment.

A key limitation is that baseline comparability could only be assessed with respect to the limited set of routinely recorded variables available across sites, namely age, gender, diagnosis group, and prior acute psychiatric service utilization. Several additional factors relevant to IHT eligibility and treatment allocation could not be verified for participants in the control group, including the availability of a suitable home environment for IHT, consent from household members or caregivers, approval from the management of care facilities (where applicable), and the absence of child welfare concerns. No harmonized baseline data were available on symptom severity, psychosocial functioning, housing context, social support, or other contextual factors that may have influenced treatment allocation and outcomes. Consequently, the matched groups may have differed in clinically relevant ways that could not be measured or adjusted for in the present retrospective analysis. Although all IHT participants fulfilled the formal indication for acute inpatient psychiatric treatment, this criterion alone cannot be taken as evidence of equivalence in baseline severity between groups.Despite our initial sample size calculation, which assumed the feasibility of including approximately 350 patients in the IHT group and an equivalent control group from inpatient treatment (total intended sample size *n* = 700), the final sample was smaller than expected. The smaller-than-anticipated sample size resulted from multiple converging factors beyond the COVID-19 pandemic. First, pandemic-related impacts were substantial: psychiatric admissions decreased by approximately 15–20% during lockdown periods, home visit protocols required extensive infection control measures that limited treatment capacity, and staff redeployments temporarily reduced IHT team availability. Second, structural and implementation factors played a key role: the IHT service at KHD commenced only in October 2020, contributing just 42 cases; meanwhile, capacity at KAU and KNK was still expanding during the study period, with KAU increasing from 14 to 20 treatment slots only in November 2020. Third, strict methodological criteria led to additional exclusions: 16% of IHT patients had treatment durations shorter than five days, often reflecting early pandemic-related discharges or initial implementation challenges. Together, these factors highlight the challenges of conducting rigorous effectiveness research during service expansion and underscore the importance of extended recruitment periods for achieving adequate statistical power in future retrospective IHT studies.

Despite the smaller-than-planned sample size, the primary outcome—hospital readmission rate—still showed statistically significant results, indicating that IHT is an effective intervention for reducing hospital readmissions.

However, the non-significant findings regarding secondary outcomes might be at least partially attributable to the reduced sample size —and, consequently, limited statistical power—relative to the initial power calculation. A detailed post-hoc power analysis revealed important constraints: with 263 matched pairs, the study achieved 85% power to detect the observed 14.4% difference in inpatient readmission rates. For the combined readmission outcome, where a non-significant 4.2% difference favoring IHT was observed (61.2% vs. 64.3%), the available sample provided only 25% power to detect this effect size. Achieving 80% power for such a small difference would have required approximately 1,100 patients per group. Similarly, analyses of day clinic utilization patterns were underpowered to detect small-to-moderate effects. These limitations suggest that some non-significant findings—particularly for combined readmission rates—should not be interpreted as evidence of equivalence but rather as inconclusive due to insufficient power. The consistent trends favoring IHT across several secondary outcomes warrant replication in larger, adequately powered studies.

Additionally, no standardized primary clinical outcome data were available on quality of life, symptom severity, psychosocial functioning, or self-efficacy over the course of treatment or follow-up. As these parameters were not systematically assessed at admission, it was not possible to compare the clinical improvement rates between the two treatment types beyond healthcare utilization outcomes.

A critical limitation of this study is the inability to assess coercive measures, their cumulative duration, and premature treatment termination rates due to technical constraints within the hospital information systems. The absence of these data affects the interpretation of our findings in several ways. First, if IHT substantially reduces the use or duration of coercive measures—as suggested by prior research—our results may underestimate its broader benefits beyond preventing readmissions. Second, differences in premature treatment termination could indicate varying levels of treatment acceptability or patient engagement that are not captured by the current utilization metrics. The missing data primarily stem from non-standardized and non-digitized documentation practices across participating sites, where heterogeneous recording systems and definitions prevented reliable data extraction. This limitation highlights the urgent need for standardized, prospective, and interoperable data systems to capture coercion-related outcomes across clinics, as these directly impact patient rights, treatment experiences, and recovery trajectories.

Another limitation was the restriction of the available data regarding the use of psychiatric and psychotherapeutic services to the hospital information system. As a result, it was only possible to estimate the initial use of outpatient psychiatric-psychotherapeutic services based on the utilization of POD services.

Finally, the selection between IHT and IT involved substantial clinical judgment beyond formal inclusion criteria. Although all IHT participants met the official requirements for acute inpatient treatment, clinicians likely favored patients perceived as more adherent for home treatment. This pragmatic approach—while appropriate for ensuring safety and feasibility—may have introduced selection bias (“cream-skimming”), potentially overestimating the relative benefits of IHT. Future research should prospectively document allocation decisions and consider designs that mitigate selection bias, such as regression discontinuity or preference-based trials.

To better evaluate the long-term effectiveness of IHT in routine clinical practice, further research is needed. This should include larger sample sizes and address both clinically and health-economically relevant questions in multiple clinics, in rural, urban and metropolitan areas that offer IHT.

## Conclusions

The present results are consistent with the notion that shifting psychiatric treatment into patients’ home environments—even for selected patients requiring acute psychiatric inpatient care—is feasible and associated with various benefits. IHT appears to reduce the risk of inpatient readmissions and the number of inpatient treatment days also on long-term. It takes significantly more time until an inpatient readmission occurs, and over this period, the findings indicate that IHT can contribute to relieving inpatient structures in the medium to long term. However, when inpatient and home-based acute treatment are considered together, overall acute psychiatric care utilization did not differ between groups over the 36-month follow-up period after discharge from the index treatment. First-time engagement with outpatient psychiatric services (such as the POD) occurs more frequently following IHT than after inpatient treatment. Taken together, these findings suggest that IHT may not reduce the overall need for acute psychiatric care, but rather redistribute care away from inpatient settings toward home-based treatment and, in urban settings such as the one studied here, toward use of a broader spectrum of psychiatric services.

In this retrospective, propensity-score matched cohort study conducted in urban psychiatric services, patients selected for intensive home treatment experienced fewer inpatient readmissions and longer time to inpatient readmission over 36 months compared with matched inpatient-treated patients. Overall utilization of acute psychiatric services did not differ, indicating a redistribution rather than a reduction of care.

These findings apply to a selected subgroup of voluntary patients with sufficient clinical stability and social resources to permit home-based acute care. They do not support generalized conclusions about all patients requiring acute psychiatric admission. IHT may represent a feasible alternative to inpatient treatment for appropriately selected patients within well-developed urban service networks, but it should be interpreted primarily as an alternative mode of delivering acute care rather than as a means of reducing total acute service use. Moreover, causal inference is limited by residual confounding. Further prospective studies with explicit eligibility tracking and broader patient populations are required.

## Data Availability

Study material and data will be available upon reasonable request.

## Abbreviations

IHT: Intensive home treatment
IEHT: Inpatient equivalent home treatment
IT: Inpatient treatment
HT: Home treatment
DC: Day Clinic
POD: Psychiatric outpatient department
IPS: Individual placement and support
FX: ICD-10 F-codes (psychiatric diagnoses)
PSM: Propensity score matching
PS: Propensity score
M: Mean
SD: Standard deviation
HIS: Hospital information system
KAU: Krankenhaus am urban
KNK: Krankenhaus neukölln
KHD: Krankenhaus kaulsdorf
